# Comprehensive Compositional Analysis of the Slit Lamp Bacteriota

**DOI:** 10.3389/fcimb.2021.745653

**Published:** 2021-11-17

**Authors:** Birgit Fritz, Edita Paschko, Wayne Young, Daniel Böhringer, Siegfried Wahl, Focke Ziemssen, Markus Egert

**Affiliations:** ^1^ Faculty of Medical and Life Sciences, Institute of Precision Medicine, Microbiology and Hygiene Group, Furtwangen University, Villingen-Schwenningen, Germany; ^2^ Food Informatics Team, AgResearch Ltd., Palmerston North, New Zealand; ^3^ Eye Center, Medical Center, Faculty of Medicine, University of Freiburg, Freiburg, Germany; ^4^ Carl Zeiss Vision International GmbH, Aalen, Germany; ^5^ Institute for Ophthalmic Research, Eberhard-Karls University, Tuebingen, Germany; ^6^ Center for Ophthalmology, Eberhard-Karls University, Tuebingen, Germany

**Keywords:** eye, hygiene, MRSA, Illumina MiSeq ^®^, microbiota (16S), 16S rRNA gene amplicon sequencing

## Abstract

Slit lamps are routinely used to examine large numbers of patients every day due to high throughput. Previous, cultivation-based results suggested slit lamps to be contaminated with bacteria, mostly coagulase-negative staphylococci, followed by micrococci, bacilli, but also *Staphylococcus aureus*. Our study aimed at obtaining a much more comprehensive, cultivation-independent view of the slit lamp bacteriota and its hygienic relevance, as regularly touched surfaces usually represent fomites, particularly if used by different persons. We performed extensive 16S rRNA gene sequencing to analyse the bacteriota, of 46 slit lamps from two tertiary care centers at two sampling sites, respectively. 82 samples yielded enough sequences for downstream analyses and revealed contamination with bacteria of mostly human skin, mucosa and probably eye origin, predominantly cutibacteria, staphylococci and corynebacteria. The taxonomic assignment of 3369 ASVs (amplicon sequence variants) revealed 19 bacterial phyla and 468 genera across all samples. As antibiotic resistances are of major concern, we screened all samples for methicillin-resistant *Staphylococcus aureus* (MRSA) using qPCR, however, no signals above the detection limit were detected. Our study provides first comprehensive insight into the slit lamp microbiota. It underlines that slit lamps carry a highly diverse, skin-like bacterial microbiota and that thorough cleaning and disinfection after use is highly recommendable to prevent eye and skin infections.

## Introduction

Surfaces with regular contact to the human body are usually contaminated with microorganisms. Most of them belong to the resident commensal skin and mucosa microbiota, but can nevertheless carry a pathogenic potential. Many studies deal with the bacterial load on daily used devices or frequently touched surfaces, which may also carry antibiotic resistant bacteria (Brady et al., 2007; Anderson and Palombo, 2009; Di Lodovico et al., 2018; Cave et al., 2019; Gohli et al., 2019). Such surfaces usually represent fomites. Fomites are of particular concern in clinical environments, as bacteria on surfaces can be transferred easily from one person to another (Weber et al., 2010; Christoff et al., 2019), promoting the spread of infectious diseases, which is particular problematic for ill or otherwise immunocompromised persons. In 2017, 8.3% of all European patients in an intensive care unit suffered from hospital acquired infections (HAI) (EDC - European Centre for Disease Prevention and Control, 2019). For surgical site infections, the percentage varied between 0.5% and 10% (EDC - European Centre for Disease Prevention and Control, 2019). It is estimated that about 20% to 40% of the HAI in intensive care units are caused by hand-to-hand transmissions (Weinstein, 1991) and that 10% of acute care patients acquire multi-drug resistant microorganisms during their stay (Cao et al., 2016).

During routine diagnostics in eye clinics, many patients are examined in a short time and often suffer from highly contagious eye infectious (Watson et al., 2018). Common HAI in ophthalmology are acute (viral and bacterial) conjunctivitis, keratitis and endophthalmitis (Wang et al., 2006), while frequent and increasing infections by multi-resistant bacteria are caused by *Staphylococcus aureus* and *Pseudomonas aeruginosa* (Schulte et al., 2020). Therefore, special hygienic attention is required for optical surfaces. Previous studies revealed a significant and diverse bacterial load on optical devices, such as microscopes (Fritz et al., 2020b), surgeons loups or surgeons eyeglasses (Graham et al., 2019; Butt et al., 2021) and reusable tonometer tips (Hillier and Kumar, 2008). They all contained significant amounts of bacteria, including many species known to cause skin and eye infections and with a potential to carry antibiotic resistances.

Slit lamps count among the most important and most often used ophthalmological devices, demanding close contact between examiner, many different patients and device surfaces. Previous studies revealed their relevant surfaces to be contaminated with bacteria, mostly coagulase-negative staphylococci, micrococci, bacilli and also *Staphylococcus aureus* (Graham et al., 2008; Sobolewska et al., 2018). However, these examinations were performed with cultivation-dependent techniques, which provide only a very limited overview on the present microbiota, as the cultivation conditions for most microorganisms are still unknown. Here, we used the cultivation-independent, molecular approach of 16S rRNA gene sequencing to analyse the bacteriota on different slit lamp surfaces in detail. Our study represents the first comprehensive analysis of the microbial contamination on slit lamps and we assume it provides a solid basis for a deeper understanding of the hygienic relevance of these widely used optical devices.

## Material and Methods

### 16S rRNA Gene Amplicon Sequencing-Based Analyses

46 slit lamps (various manufacturers) stemming from the Center of Ophthalmology, University Hospital Tuebingen (hereinafter called Tertiary Center 1 – TC1), Germany (n = 29) and the Eye Center, Medical Center, University of Freiburg (hereinafter called, Tertiary Center 2 – TC2), Germany (n = 17) were swab-sampled in October 2020, during routine patients examinations within an unannounced audit. TC1 and 2 were chosen as they are among the largest tertiary care facilities for specialized, consultative, ophthalmological health care in our region and allowed for sufficient sample material.

**Figure 1:**
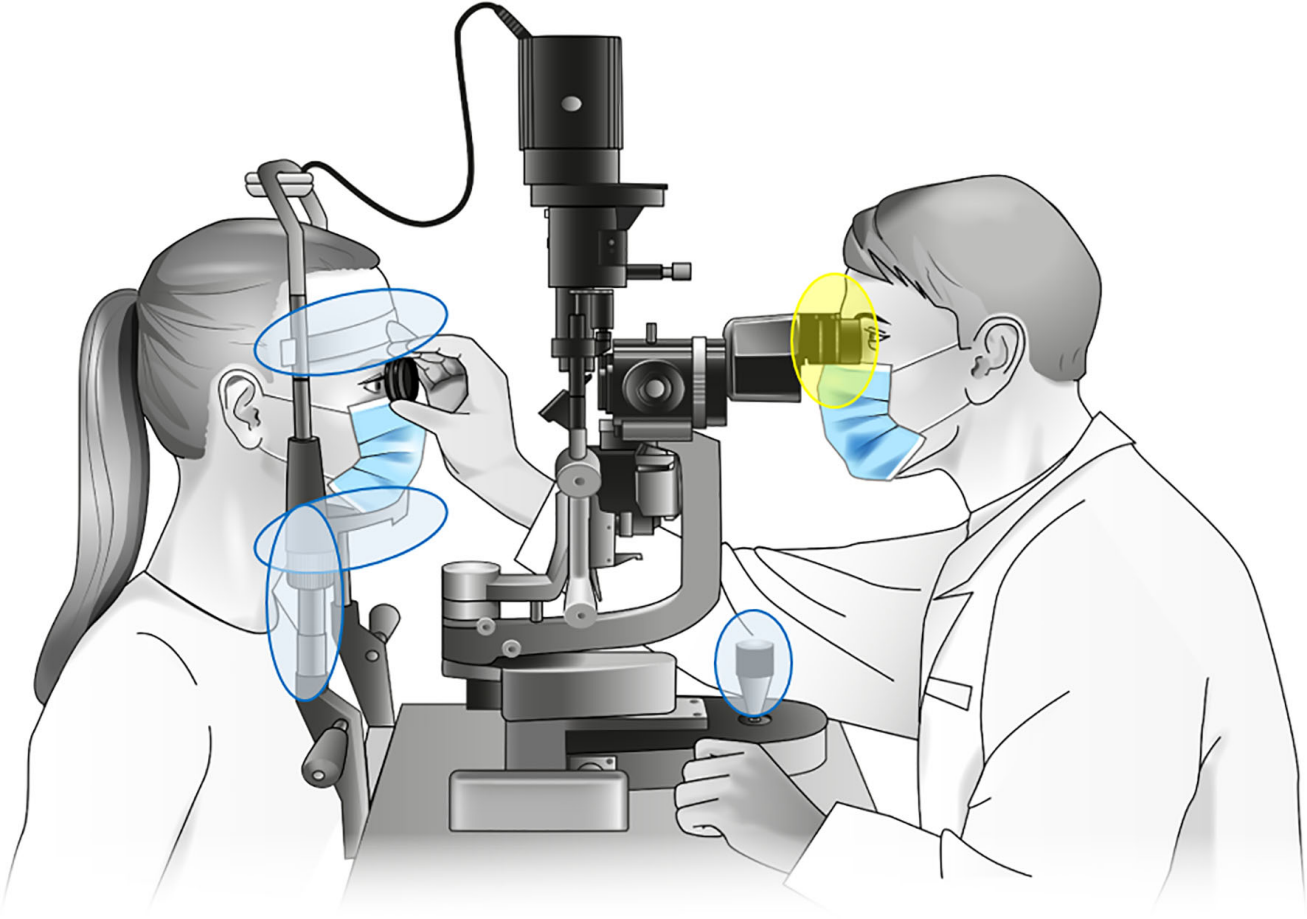
Sampled parts of the investigated slit lamps. Samples designated “contact area” were taken from headrest, headband, joystick and handholders (marked blue), with probable microbial transmission between patient and physician *via* the handrails. Samples designated “oculars” were taken from the lenses and plastic eyecups (marked yellow). Slit lamp graphic: Own illustration.

Patient throughput ranged between 4 and 200 patients per day within the respective rooms (median ± SD: 81 ± 47.1). Predominately rooms with a high patient throughput were chosen, as a high occupancy rate was suspected to lead to higher bacterial load and diversity. Rooms with a low patient throughput were sampled as reference. The examination rooms were shared between 1 and 20 physicians per day (median ± SD: 6 ± 4.6). To ensure comparability with previous studies (Sobolewska et al., 2018; Fritz et al., 2020b), the following regions of slit lamps were sampled, resulting in two samples per device: The ‘oculars’ (lens and plastic eyecup) as regions in close proximity to the physicians eyes and the surfaces with direct skin contact, such as the joystick, the handrail, the headrest and the headband (pooled as ‘contact area’, **Figure 1**). In both clinics, all slit lamp contact areas were claimed to be wipe disinfected between different patients. More comprehensive cleaning data were obtained from TC1. Here, slit lamps were in addition cleaned carefully either three times a day, once a day or weekly. All relevant metadata details are provided in the **Supplementary Table 1**.

All surfaces were sampled in a meandering pattern using dry, sterile Puritan Hydra Flock Swabs (Puritan Diagnostics LLC, Maine, USA). After sampling, swab heads were broken off into RNA/DNA shield tubes with beads (Zymo Research, Freiburg, Germany) and stored at room temperature until further processing.

### DNA Extraction

For cell disruption, collected swab heads were treated in a FastPrep 24 instrument (MP Biomedicals LCC, Santa Ana, CA, USA) by five rounds of bead beating for 1 min at 6.5 ms^-1^ and then placed on ice for 1 min. DNA was then extracted and purified with the ZymoBIOMICS DNA Miniprep Kit (Zymo Research) following the manufacturer’s instructions with slight modifications: After 2 min of incubation at room temperature, the DNA was eluted with 50 µl of 60°C warm, DNA-free water. The flow through was reloaded onto the same filter, incubated for 1 min and centrifuged again. The purified DNA was stored at -20°C until further analyses.

### Preparation of Controls

To better evaluate the community composition analysis process and probable contaminations, positive (mocks) and negative controls were carried out along the experiment. The Skin Microbiome Whole Cell Mix (ATCC MSA-2005, LGC Standards GmbH, Wesel, Germany) was used as a mock community standard, covering a typical part of the human skin bacterial community. The mock community consisted of 6 typical skin bacterial species in equal total cell abundances (*Acinetobacter johnstonii* ATCC 17090, *Corynebacterium striatum* ATCC 6940, *Micrococcus luteus* ATCC 4698, *Cutibacterium acnes* ATCC 11828, *Staphylococcus epidermidis* ATCC 12228 and *Streptococcus mitis* ATCC 49456, 16.7% each). The standard was prepared according to the manufacturer´s recommendations (American Type Culture Collection, 2018), so that the final suspension contained about 1.2 x 10^8^ cells/vial (± 1 log). As negative (blank) control, two sterile swabs were processed independently as described above.

### Library Preparation

For construction of amplicon libraries, primers Bact-0341f (5’-CCTACGGGNGGCWGCAG-3’) and Bact-0785r (5’-GACTACHVGGGTATCTAATCC-3’), covering the V3-V4 region of the bacterial 16S rRNA gene, were used. We chose this primer pair as it is widely used in many microbiome studies (Klindworth et al., 2013; Thijs et al., 2017; Illumina, 2019; Mancabelli et al., 2020) also with regard to skin (Castelino et al., 2017) and oral microbiota (Zheng et al., 2015), however sometimes with slight modifications. All primers contained an additional adapter sequence tail (Forward overhang: 5’ TCGTCGGCAGCGTCAGATGTGTATAAGAGACAG; Reverse overhang: 5’ GTCTCGTGGGCTCGGAGATGTGTATAAGAGACAG), yielding a final PCR product of ~ 529 bp.

Samples were processed in duplicates. Triplicates were performed, if the gel electrophoresis showed only weak bands.

All samples were amplified in a total reaction volume of 25 µl using 3 µl of template DNA as specified elsewhere (Fritz et al., 2020b). The PCR conditions were as follows: 98°C (3 min) initial denaturation, followed by 98°C (30 s), 55°C (30 s), 72°C (45 s), and a final extension at 72°C for 2 min using 35 cycles. DNA amplicons were checked by standard 0.8% agarose gel electrophoresis. With each batch, no-template control reactions were included. Diluted (1:100) DNA from overnight cultures of *Escherichia coli* K12, extracted as described above, was used as template for the positive controls. Clean-up of the PCR products using Agencourt AMPure XP Beads (BeckmanCoulter Inc., Krefeld, Germany), followed by annealing of the dual-index barcodes from the Nextera XT Index Kit v2 Set B (Illumina Inc., San Diego, USA), was performed as described previously (Fritz et al., 2020b). The cleaned libraries were quantified using a Qubit 2.0 Fluorometer (Thermo Fisher Scientific, Karlsruhe, Germany), while the final quality check was performed with a Bioanalyzer 2100 Instrument with the DNA High Sensitivity Kit (both Agilent Technologies Deutschland GmbH, Waldbronn, Germany).

### Sequencing

The libraries were adjusted to 4 nM (with 10 mM Tris buffer, pH 8.5), combined with 30% PhiX control (Illumina Inc.) and finally diluted to 4 pM. Sequencing was performed on an Illumina MiSeq platform using the MiSeq Reagent Kit v3 (600 cycle) (Illumina Inc.) with a quality score ≥ 30 and default settings.

### Quantitative Real-Time PCR Detection of MRSA

The obtained DNA extracts were used for a quantitative real-time PCR (qPCR) approach targeting the *mecA* gene of MRSA as described by Huletsky and collegues (Huletsky et al., 2004). The *mecA* gene causes methicillin resistance in *S. aureus* and is part of the staphylococcal cassette chromosome *mec* element (SCCmec), an important mobile genetic element of staphylococci.

The *S. aureus*-specific primer Xsau325f (*5’-* GGATCAAACGGCCTGCACA-3’) and the resistance specific pr-imer SSCmec_mecii574r (*5’-* GTCAAAAATCATGAACCTCATTACTTATG-3’) were used at 0.4 µM with 1x Light Cycler 480 SYBR Green Master mix I (Roche Molecular Systems Inc., Mannheim) and 1 U Uracil-DNA Glycosylase (Thermo Fisher Scientific) in a final volume of 20 µl. The mixture was amplified on an Roche LightCycler 480 instrument (Hoffmann-La Roche Ltd, Basel, Switzerland) using the following thermal profile: 50°C (2 min), 95°C (5 min), 45 cycles of 95°C (10 sec), 60°C (20 sec), 72°C (30 sec).

Absolute quantification analysis using the 2^nd^ derivative maximum method was performed followed by a melting curve, applying the ‘*Tm*-calling’ method with default settings. The limit of detection (LOD) was set to a *cp* = 33 using a standard logarithmic serial dilution from Methicillin-resistant *Staphylococcus aureus* EDCC 5246 (DSM 28766). As negative controls, water-template controls were included, as well as the antibiotic sensitive strains *S. aureus* 209 (DSM 799) and *S. aureus* Wichita (DSM 2569). The latter shows only limited antibiotic resistance. Control DNA was extracted from 48 h bacterial cultures as described above and diluted 1:10. To control, if the used staphylococci indeed show antibiotic sensitivity, all strains were plated on Tryptic Soy Agar (TSA; Carl Roth) and Oxoid Brilliance MRSA 2 Agar (Thermo Fisher Scientific). The Brilliance MRSA 2 Agar contains an antibiotic cocktail including Cephalosporin, whereby MRSA grows as blue colonies (Veenemans et al., 2013).

### Bioinformatics and Statistics

All sequences were processed with QIIME 2 – 2020.6 (Quantitative Insights Into Microbial Ecology) (Bolyen et al., 2019). Raw sequence data were imported and demultiplexed using the cassava 1.8 paired-end and demultiplexed FASTQ format. The paired end sequences were joined, quality filtered, denoised and chimera-checked using the q2-dada2 pipeline (–p-trunc-len-f 300 –p-trunc-len-r 257 trim-left-f 0 –p-trim-left-r 0) (McDonald et al., 2012; Callahan et al., 2016). Sequence variant data, resulting from the q2-dada2 pipeline, were then referred to as amplicon sequence variants (ASVs).

For taxonomic assignment, the machine-learning based q2-feature-classifier was trained at a similarity threshold of 99% with q2-scikit-learn (Pedregosa et al., 2011; Bokulich et al., 2018) by the Bact-0341f/Bact-0785r region (V3-V4) of the SILVA 132 database (Quast et al., 2013), followed by taxonomy based-filtering of ASVs classified as mitochondria or chloroplasts.

Due to the sparse compositional nature of microbiome data, beta diversity metric was analysed using robust Aitchinson distances *via* the q2-deicode plugin (Aitchison et al., 2000; Martino et al., 2019). Calculations were performed on the raw count table. Samples with less than 10 features and less than 1000 reads were removed. Statistical differences between the factors ‘location’ (TC1/TC2) and ‘type’ (oculars/contact areas) were performed using the ‘beta-group-significance’ function employing (pairwise) permutational multivariate analysis of variance (PERMANOVA, 999 permutations) with Benjamini-Hochberg p-value correction. For visualisation, compositional principal component analysis (PCA) biplots were created using the emperor biplot function.

Further statistical analyses and graphical visualizations for the sequencing analyses were performed in R 4.0.5 using the packages ‘phyloseq’ (McMurdie and Holmes, 2013), ‘vegan (version 2.5-7)’ (Oksanen et al.), ‘coin’ (Hothorn et al., 2008), ‘tidyverse’ (Wickham, 2009) and ‘qiime2R’ (Bisanz, 2019). Figures were created in R using ‘ggplot2’ (Wickham, 2009).

Correlation of microbial composition between the sample sites at TC1 and TC2 were assessed by procrustes rotation analysis comparing PCA scores, using the ‘procrustes’ function from the ‘vegan’ package for R (Peres-Neto and Jackson, 2001; Mardia et al., 2003). The significance of the correlation between samples sites was analysed using the ‘protest’ function from ‘vegan’, using 10.000 permutations.

To identify differentially abundant taxa between the covariates ‘location’ and ‘type’, analysis of compositions of microbes with bias correction (ANCOM-BC; R-package ‘ancombc’) (Lin and Peddada, 2020; Edslev et al., 2021) was performed on untransformed and unrarefied counts, including only the 30 most abundant taxa. As there is a bias within the sequence fractions of all samples, this method estimates the unknown sampling fractions and corrects their bias, while normalizing the observed microbial abundance data (Lin and Peddada, 2020). Results are p-values for multiple testing with Benjamini-Hochberg adjustment.

To determine any differences between the communities on the respective surfaces and between the locations, alpha diversity metrics (observed, evenness, faith’s phylogenetic diversity and shannon) were calculated using the R-packages ‘phyloseq’, ‘microbiome’ (Lahti et al., 2017) and ‘picante’ (Kembel et al., 2010). For comparative analysis of the diversity indices among the different sample sites, a non-parametric Kruskal-Wallis-Test (Hollander et al., 2014) was performed. A Wilcoxon-rank-sum-test (Hollander et al., 2014) for unpaired samples was calculated to evaluate statistical differences between the two locations TC1 and TC2. Both tests were performed with Benjamini-Hochberg multiple test correction. All metadata are provided in the **Supplementary Table 1**. Further data, such as the unrarefied ASV table and the taxonomic assignments, can be obtained from the corresponding author upon reasonable request.

## Results

### Sequencing Results

High throughput sequencing from 96 samples (91 slit lamp samples, 3 mock samples, 2 negative controls) yielded 467842 chimera-filtered sequences after the dada2 pipeline, with a mean of 5377 sequences per sample. 9 samples (2 TC1 slit lamp samples, 6 TC2 slit lamp samples and 1 blank control sample) did not yield enough sequences and were removed after the q2-dada2 pipeline for downstream analyses. The remaining samples were rarefied for the calculation of relative abundances using R to a level of 1056 sequences for even sampling depth (seed: 1121983). After removal of singleton taxa, we identified 3369 ASVs from all slit lamp samples. The taxonomic assignment of these ASVs revealed 19 bacterial phyla, 42 classes, 105 orders, 210 families and 468 genera across all slit lamp samples.

### Taxonomic Composition at Different Locations

**Figure 2:**
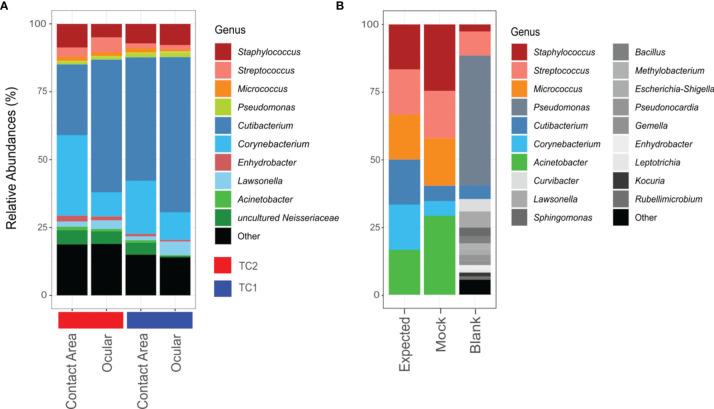
Bar chart of relative abundances of ASVs classified on genus level. Taxa with relative abundance of less than 1% were collectively summarized as ‘Other’. **(A)** Taxonomic composition of the slit lamp bacteriota from TC1 (Tertiary Center) and TC2 respectively and the respective sample sites (contact area – TC1: n = 28; ocular – TC1: n = 28; contact area - TC2: n = 14; ocular – TC2: n = 13). **(B)** Composition of mock taxa and controls (expected = expected mock abundances; mock = mock standard using Bact-0341f/Bact-0785r primers; blank = blank negative control).

**Figure 2A** (and **Supplementary Figure 5**) provide relative abundances to get an overview of the community composition. However, for the comprehensive downstream analyses, we used methods that are not based on relative abundances, due to the compositional nature of sequencing data (Gloor et al., 2017).

According to the phylogenetic classification, most of the reads were affiliated with only 10 genera accounting for about 80% of all taxa, with *Cutibacterium* (TC1: 51%; TC2: 38%), *Corynebacterium* (TC1: 15%; TC2: 19%) and *Staphylococcus* (TC1: 8%; TC2: 7%) being the most frequent representatives.

Using the V3-V4 region specific Bact-0341f/Bact-0785r primers, all genera from the mock community were identified correctly, but in slightly varying relative abundances (**Figure 2B**). *Corynebacterium* and *Cutibacterium* were rather underrepresented, while *Acinetobacter* and *Staphylocoocus* seem to be slightly overestimated in their relative abundance. In case of the negative controls, only one out of two unused swabs yielded enough sequences for downstream analysis, which suggests contamination predominantly with *Pseudomonas* specie*s*.

### Diversity Analyses

As the relative abundances of bacterial taxa differed between the two sampling sites (contact area, oculars) and the two locations (TC1, TC2), alpha diversity metrics were calculated. Diversity tended to be higher in TC2 with regard to the observed, shannon and faith-fd metrics, but no statistically significant differences could be detected, neither between TC1 and TC2 nor between contact areas and oculars (**Supplementary Figure 1**) or the cleaning intervals, three times a day, once a day or weekly, within TC1 (**Supplementary Figure 2**) or occupancy of physicians (**Supplementary Figure 4**).

Compositional beta diversity analysis also revealed no statistical significant differences between the locations (PERMANOVA p_adjust_-value > 0.05) and the contact areas (PERMANOVA p_adjust_-value > 0.05). However, some samples from TC1 tended to cluster apart from TC2.

**Figure 3:**
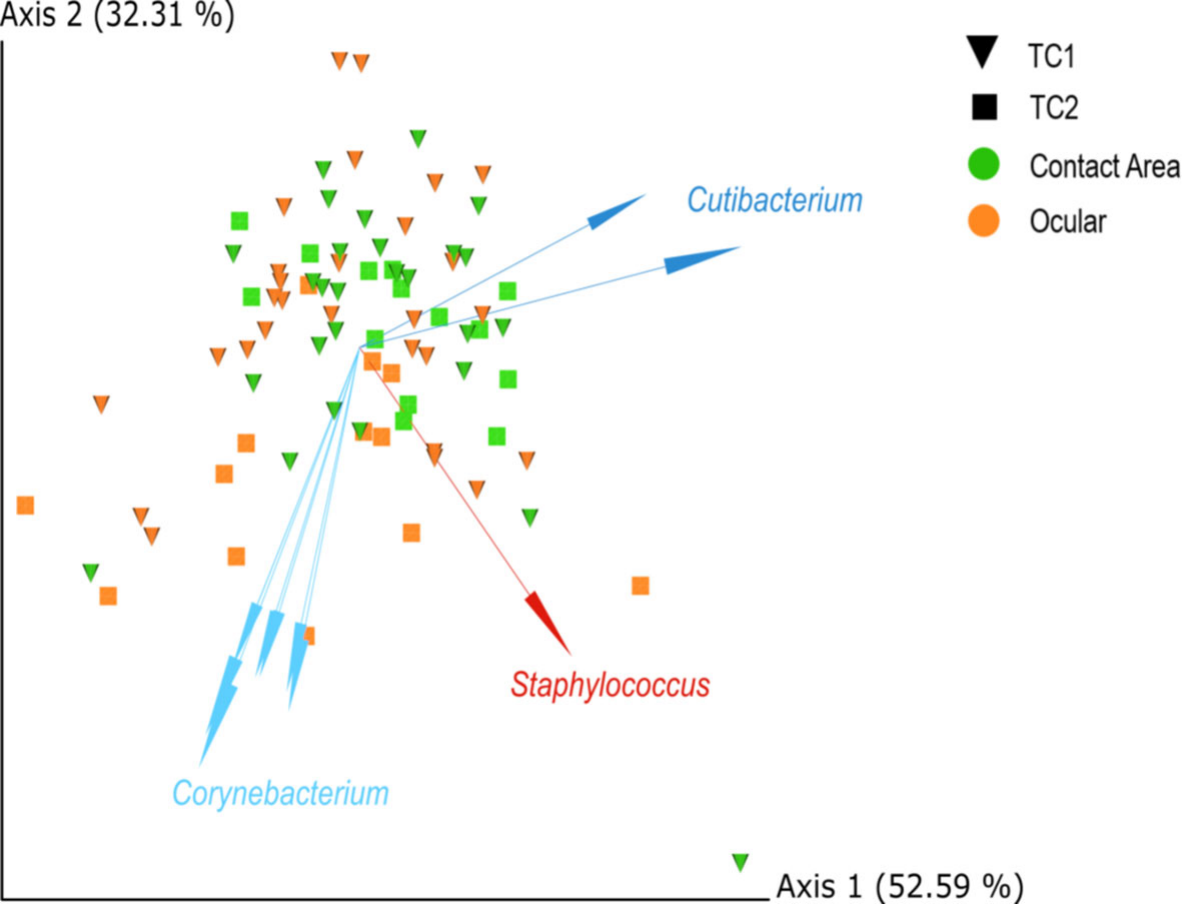
PCA biplots of centred-log-ratio (clr)-transformed data for Location and Type, based on robust Aitchinson distances. Arrows indicate important log-ratios between features that strongly influenced the principal component axis. Squares = TC1, cones = TC2, orange = ocular samples and green = contact area samples; TC, Tertiary Center.

The biplot (**Figure 3**) highlights the taxa which drive the placement of the samples in the plot and strongly influence the PCA-axes. Some separation of samples from TC1 and TC2 are presumably driven by differences within the taxonomic composition, especially by corynebacteria.

No statistically significant influence of the patient throughput and the occupancy of physicians for the different rooms was detected (**Supplementary Figures 3**, **4**, **5B, C**), although alpha diversity measures suggest a slightly higher diversity on slit lamps in rooms with low occupancy rates at TC1.

**Figure 4:**
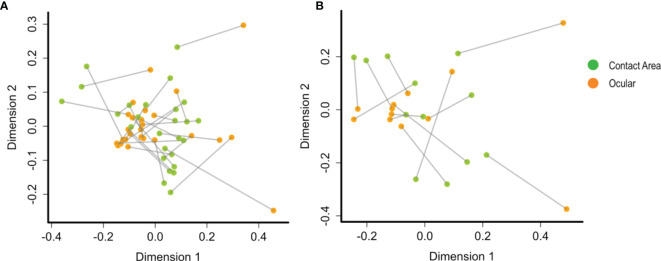
PCA plots by procrustes analysis between the ocular and contact area data sets using Bray-Curtis distances and 10.000 iterations. At **(A)** TC1 (contact area: n =28; ocular: n = 28) and **(B)** TC2 (contact area: n = 14; ocular: n = 13). Every data point represents a single slit lamp sample coloured by sample site (orange = ocular samples, green = contact area samples). Lines connect the same slit lamp samples (Contact area – TC1: n = 28 ocular – TC1: n = 28; contact area - TC2: n = 14; ocular – TC2: n = 13). TC, Tertiary Center.

To determine the community congruency between the ocular and contact area samples, we used procrustes analysis, which compares the microbiome on the two sample sites of each slit lamp. However, no statistically significant association could be detected. Nevertheless, a correlation between microbial compositions at the two sampling sites from the same slit lamp instrument is suggested, since many sample pairs stemming from the same instrument are located in relatively close proximity within the PCA plot (**Figure 4**), especially at TC1.

**Figure 5:**
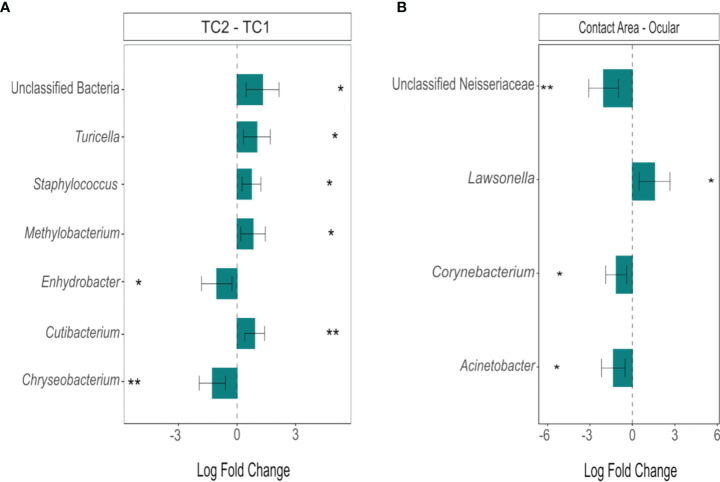
Differentially abundant genera between **(A)** the two locations (TC2 – TC1) and **(B)** the sample types at TC1 (contact area – ocular), calculated using ANCOM-BC. Bars represent the estimated log-fold change (natural log) of genera between the selected conditions, error bars represent the 95% confidence intervals. Asterisks mark a statistically significant difference between the conditions (*p_adj_ < 0.05; **p_adj_ < 0.01), based on two-sided Chi-square tests using W with Benjamini-Hochberg correction; TC, Tertiary Center.

However, differential abundance analysis (**Figure 5**) revealed the taxa with significant differences between the two locations: Two taxa were more abundant in TC2 (*Enhydrobacter*, *Chryseobacterium*; p_adj_ < 0.05), whereas five groups/genera (unclassified bacteria, *Cutibacterium*, *Turicella*, *Methylobacterium*, *Staphylococcus*, p_adj_ < 0.05) were enriched at the slit lamps at TC1. At TC1, four genera showed significant differences between the two sample sites (p_adj_ < 0.05): *Unclassified Neisseriaceae, Lawsonella, Corynebacterium* and *Acinetobacter*. Except *Lawsonella*, all genera were enriched at the contact areas. For the slit lamps at TC2, no significant difference in community composition between oculars and contact areas were detected.

### Quantitative Real-Time PCR Results for MRSA

MRSA strain *S. aureus* EDCC 5246 grew as blue colonies on Brilliance Oxoid MRSA2 agar after 48 h at 37°C, whereas the other *S. aureus* strains did not thrive on the respective plates.

Genomic DNA from *S. aureus* EDCC 5246 was used as standard and positive control in the assay. DNA was prepared and 1:10 diluted from 48 h old cultures that yielded a DNA concentration of 1.94 ng/µl resulting in a mean *cp*-value of 17 and a melting temperature (*T_m_*) of 79.8°C. The detection limit (LOD) was determined at a *cp* -value of 33, corresponding to a 1:1000 dilution. Although higher dilutions also showed *cp*-values, these curves were in too close proximity to signals from the non-MRSA-strains.

The antibiotic sensitive *S. aureus* 209 showed *cp* -values > 36 and the antibiotic susceptible *S. aureus* Wichita *cp* -values > 35. Water-template controls resulted in *cp* values > 40 or showed no signals. Only one sample from TC2 (contact area) showed relevant *cp*-values of 35 and a melt-peak at 80°C, which is close, but still below the detection limit, respectively close to the MRSA *S. aureus* Wichita. In conclusion, MRSA DNA was below the detection limit for all investigated samples.

## Discussion

Many regularly touched surfaces represent fomites (Rusin et al., 2002; Gerba et al., 2016; Di Lodovico et al., 2018). Their microbial load is problematic especially in clinical environments (Weber et al., 2010; Graham et al., 2019), where it contributes to hospital acquired infections and particularly threatens immunocompromised patients. This study focuses on slit lamps, optometric devices widely used in medical eye facilities, characterized by surfaces with close contact to the examiner and many different patients. Although relations between shared optical devices and eye infections have long been suspected (Olcerst, 1987), knowledge about the bacterial contamination of slit lamps is scarce. Previous cultivation-based examinations, (Graham et al., 2008; Sobolewska et al., 2018) of slit lamps proved the presence of human skin bacteria, however with max. amounts of 3 CFU/24 cm^2^ at a relatively low concentration, which is at least 2 to 3 log scales less, than the bacterial load on similar devices, such as spectacles (Fritz et al., 2018) and microscope oculars (Olcerst, 1987; Fritz et al., 2020b). Recent molecular studies (Fritz et al., 2020b; Fritz et al., 2020a) of the microbiota on such surfaces, revealed a high bacterial diversity stemming from human skin, mucosa and the environment, and including potential pathogens. Our study significantly increases knowledge about the bacterially diverse communities on slit lamp surfaces and their hygienic relevance. It completes the picture, when evaluating the microbial contamination of slit lamps, as previous studies have been limited to cultural-dependent analyses alone. Molecular methods better account for anaerobic, slow-growing or yet uncultured bacteria and therefore provide a more comprehensive insight. Following recent suggestions (Gloor et al., 2017; Martino et al., 2019), our bioinformatic analyses also considers the compositional nature of sequencing data.

Clearly, also sequencing-based methods can be biased, e.g. by discriminating certain groups of organisms due to primer selectivity (Kong, 2016; Kong and Segre, 2017; Zeeuwen et al., 2017; Escapa et al., 2018; Knight et al., 2018). Therefore, we evaluated the used primer set on a standard skin-mock community (**Figure 2B**). It became apparent, that all typical skin bacteria were covered comprehensively, while the primers also produced good quality PCR products (data not shown). However, as all sequencing data presented here are DNA-based, they do not reveal if the detected bacterial taxa were alive or dead.

As negative controls, unused swabs (blank) were processed to identify potential contaminants. In one out of two swabs, DNA from bacteria probably stemming from water, environmental and human sources could be detected, dominated by *Pseudomonas.* It is known that even small amounts of contaminating DNA can become overrepresented using PCR-based methods and influence the community composition of a sample (Karstens et al., 2019). Generally, the identified genera in the blank control were reported before as typical contaminants from analysis kits, PCR reagents or water in NGS-experiments (Salter et al., 2014), or as representing just sequencing errors. Therefore, it is recommended to remove all sequences below a defined relative abundance threshold from further analysis (Karstens et al., 2019). In accordance with the results from the mock samples, we set this threshold at 1% relative abundance. Therefore, it is safe to assume that contaminants from exogenous sources are negligible here.

Our results show bacterial contaminations on 83 out of 91 investigated slit lamp samples, as these yielded enough sequences for downstream analyses. Based on the relative abundances of the bacterial taxa and alpha diversity measures, the bacteriota composition was largely similar between the two investigated locations and the two different sample sites. A few taxa differed in read abundance between TC1 and TC2, which may reflect the individuality of the patient´s and/or doctor´s skin microbiota.

Predominantly, we identified cutibacteria, corynebacteria and staphylococci on the slit lamps. This is in line with previous findings for spectacles and microscope oculars, where we found largely the same dominant taxa. We could also prove the presence of staphylococci, which matches the cultivation-based detection of coagulase-negative staphylococci by Sobolewska et al. (Sobolewska et al., 2018). While they identified staphylococci as the most frequent bacteria on slit lamps, we found this genus in lower proportions, which may be due to the use of different methods. Nevertheless, their presence is important, as staphylococci, along with streptococci, are among the most common bacteria to cause bacterial conjunctivitis and keratitis (Watson et al., 2018).

In general, many of the identified bacterial taxa are common colonizers of the eye surface and the lid margins, such as *Staphylococcus*, *Cutibacterium*, *Corynebacterium* and *Pseudomonas* (Grzybowski et al., 2017; Delbeke et al., 2021). Under normal conditions, these commensals may also contribute to eye surface health and immunity (Kugadas and Gadjeva, 2016; Cavuoto et al., 2019), however, if in dysbiosis they may lead to severe eye infections. In addition to the previously mentioned taxa, the relatively most abundant identified genera also included *Streptococcus, Neisseria* and *Enhydrobacter.* All these genera are known to comprise several species with the potential to cause eye infections (cf. **Supplementary Table 2**). Although our sequencing data do not allow a reliable identification on species or strain level, they nevertheless suggest a considerable pathogenic potential of the investigated surfaces.

All frequently identified genera are also associated with human skin, mucosa or the environment (Seifert et al., 1997; Fernández-Natal et al., 2008; Humbert and Christodoulides, 2019; Delbeke et al., 2021) and have been reported in the context of eye and nosocomial infections (Kuriyan et al., 2017; Wong et al., 2017; Humbert and Christodoulides, 2019).

While the oculars of slit lamps are predominantly used by only a few physicians, surfaces such as headrest, headband and handholders are touched by many different patients, as well as by the examiner. However, alpha diversity analysis did not detect any statistical significant differences of the bacteriota composition on the two sampled sites, nor between samples with different patient and physician occupancies or cleaning intervals.

Procrustes analysis suggested a relatedness of microbial community compositions at the two sampling sites from the same slit lamp instrument. However, this association was not found statistically significant, which may be a function of the relatively low number of sampled instruments. Nevertheless, our data allow careful speculations that bacteria between ocular and contact areas are exchanged, putting emphasis on hygienic cleaning of the ocular area after use.

Evaluation of the different cleaning intervals in TC1 did not reveal statistically significant differences in bacterial community composition. As the contact areas were wipe disinfected on a regular basis, frequent contact by different persons obviously could not contribute to an increased bacterially diverse community for both locations and sampling sites. Notably, our sampling took place during the COVID-19 pandemic, which was accompanied by special hygienic measures, such as more frequent cleaning and disinfection as well as rigorous wearing of face-masks. This might have influenced bacterial load and diversity on the investigated surfaces. Nevertheless, microbial transfer between skin, eyes and slit lamp surfaces might take place by touching, but also breathing or direct eye or eyelashes contact. In view of the overall great bacterial diversity detected here, strict hygiene measures are definitely required.

Multi-drug resistances are of particular hygiene concern. Methicillin-resistant *Staphylococcus aureus* (MRSA) is known to be highly prevalent in hospital environments (Boucher and Corey, 2008), but also on daily used and shared devices (Gerba et al., 2016), showing a high transmission efficiency (Del Campo et al., 2019). Previous 16S rRNA gene sequencing studies (Fritz et al., 2020b; Fritz et al., 2020a) identified many staphylococci on optical surfaces, while its known, that several staphylococci comprise antibiotic resistant strains (Ventola, 2015), such as MRSA. Our qPCR analysis did not reveal the presence of MRSA in any sample. We used a single primer pair published by Huletsky et al. (Huletsky et al., 2004), which covers a variety of MRSA strains. A multiplex assay would expand the detection spectrum and could be considered for further studies. However, since many of the identified taxa comprise species known to carry (multiple) antibiotic resistances, this topic remains challenging. Further studies might also include a metagenomic approach, such as whole genome shotgun sequencing (WGS) to allow a more comprehensive detection of resistant genes and virulence factors. Furthermore, if enough reads are generated, WGS can provide a broader and more accurate resolution of microbial diversity, especially for less abundant taxa (Ranjan et al., 2016). However, as WGS does not rely on PCR amplification, it requires a larger amount of input DNA compared to 16S rRNA amplicon sequencing, which often turns out to be a limiting factor. Finally, polyphasic studies (such as (Fritz et al., 2020b), involving cultivation-based as well as molecular techniques might be useful to better discriminate living from dead taxa, the latter of which are surely less important from a hygienic point of view.

Beside bacteria, viruses, such as Adenovirus or Herpes simplex, would be interesting study subjects, as many viruses cause severe eye infections (Yoshikawa et al., 2001; Azher et al., 2017; OYong et al., 2018). A significant viral load on slit lamps may be possible, as studies showed that they can remain infectious on environmental surfaces for considerable time periods (Kramer et al., 2006; Ganime et al., 2014).

## Conclusion

We were able to show that slit lamps carry a broad diversity of bacteria and therefore might be associated with ophthalmic diseases. Our study significantly extends previous findings about their bacterial load by applying molecular, cultivation-independent techniques. It provides a solid and comprehensive basis for a deeper understanding of the hygienic relevance of these widely used medical devices. We identified many bacterial genera of human skin, eye or mucosa origin, known to comprise species to cause skin and eye infections, such as staphylococci or streptococci. Even if wipe cleaning is performed regularly between each patient, also the disinfection of the oculars, mostly used by the physicians, should be considered. Clearly, slit lamps represent fomites and proper disinfection of all contact surfaces is important to secure the health of patients and examiners.

## Data Availability Statement

The datasets presented in this study can be found in online repositories. The names of the repository/repositories and accession number(s) can be found at: https://www.ebi.ac.uk/ena, PRJEB45031.

## Author Contributions

BF designed the experiments, took the samples, performed qPCR analyses, sequenced the samples and analyzed the data. EP prepared sequencing. FZ and DB provided access to the samples. SW, FZ, and ME performed project administration and supervised the work. BF and ME wrote the manuscript. ME supervised and conceptualized the work. WY assisted with the ancom-bc and procrustes analysis. All authors edited and approved the final manuscript. All authors contributed to manuscript revision, read, and approved the submitted version.

## Funding

This study received funding from a grant of the German Federal Ministry of Education and Research (https://www.bmbf.de/en/index.html; project CoHMed - Connected Health in Medical Mountains; subproject ‘FunktioMed’, grant number 13FH5I02IA) and from Carl Zeiss Vision International GmbH (https://www.zeiss.com/corporate/int/home.html). The funders were not involved in the study design, collection, analysis, and interpretation of data, the writing of this article or the decision to submit it for publication.

## Conflict of Interest

Author WY was employed by company AgResearch Ltd. Author SW was employed by company Carl Zeiss Vision International GmbH.

The remaining authors declare that the research was conducted in the absence of any commercial or financial relationships that could be construed as a potential conflict of interest.

## Publisher’s Note

All claims expressed in this article are solely those of the authors and do not necessarily represent those of their affiliated organizations, or those of the publisher, the editors and the reviewers. Any product that may be evaluated in this article, or claim that may be made by its manufacturer, is not guaranteed or endorsed by the publisher.

## Abbreviations

ASV, Amplicon Sequence Variant; HAI, hospital acquired infections; TC, Tertiary Center; SCCmec, staphylococcal cassette chromosome; PCA, principal component analysis; ANCOM-BC, analysis of compositions of microbes with bias correction; qPCR, quantitative PCR; MRSA, methicillin-resistant Staphylococcus aureus; PERMANOVA, permutational multivariate analysis of variance; QIIME, Quantitative Insights Into Microbial Ecology.
