# Supplementary material for: Comprehensive Compositional Analysis of the Slit Lamp Bacteriota
Source: Front Cell Infect Microbiol. 2021 Nov 17;11:745653. doi: 10.3389/fcimb.2021.745653 (PMC8635730; doi:10.3389/fcimb.2021.745653)
Supplement: Supplementary file 1 [file DataSheet_1.pdf]

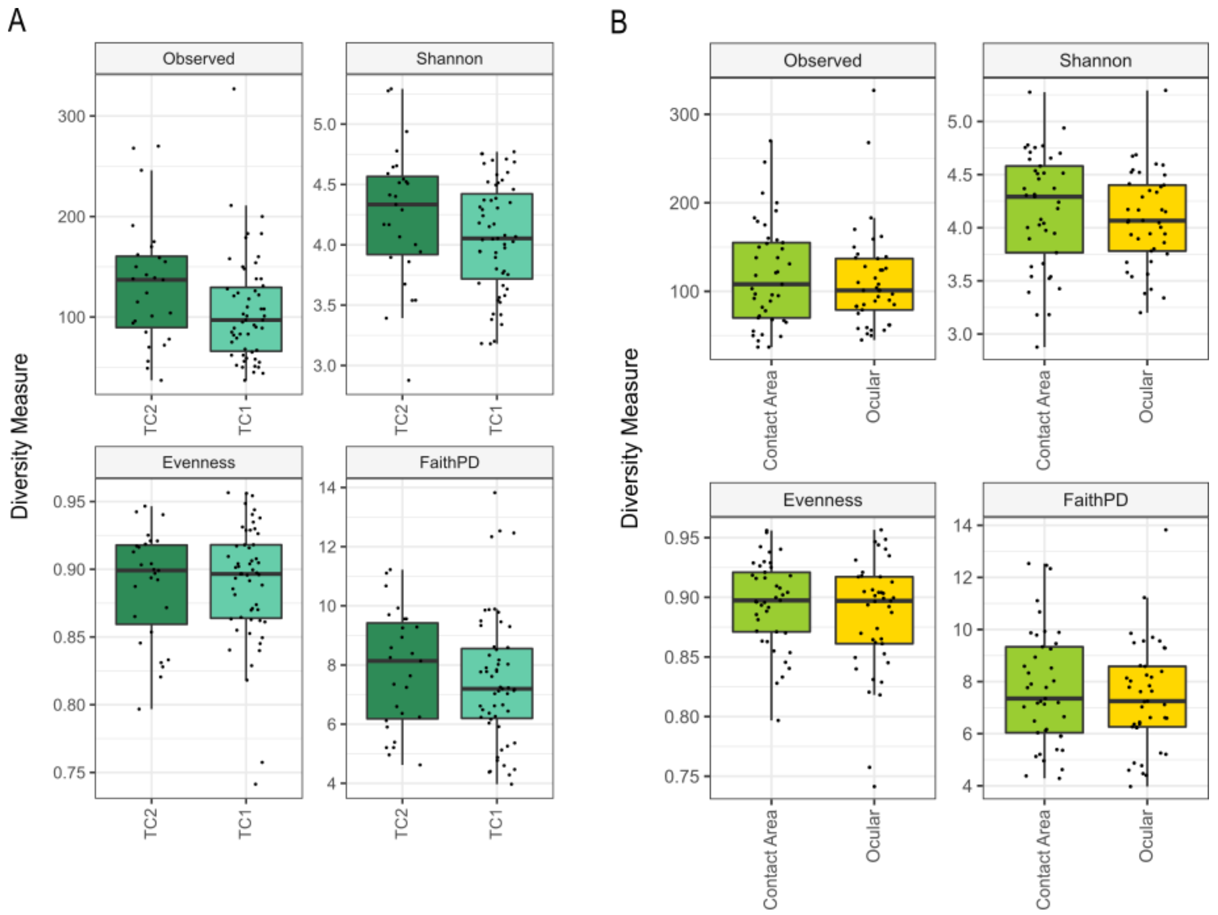

Supplementary Figure 1. Comparison of alpha diversity measures between locations (A) (TC1: n = 56, TC2: n = 27) and the overall sample sites (B) (Contact Area: n= 45, Oculars: n = 46). Differences are shown by four indices (observed taxonomic units, Shannon, Pielou's Evenness, Faith PD diversity index). Points represent individual samples. Displayed are the median, the 25% and 75% quartiles and outliers. Whiskers represent the lowest and highest microbial counts within the 1.5-fold of the interquartile range (IQR) (the 25% and 75% quartile). No differences were found statistically significant ($p > 0.05$). TC = Tertiary Center.

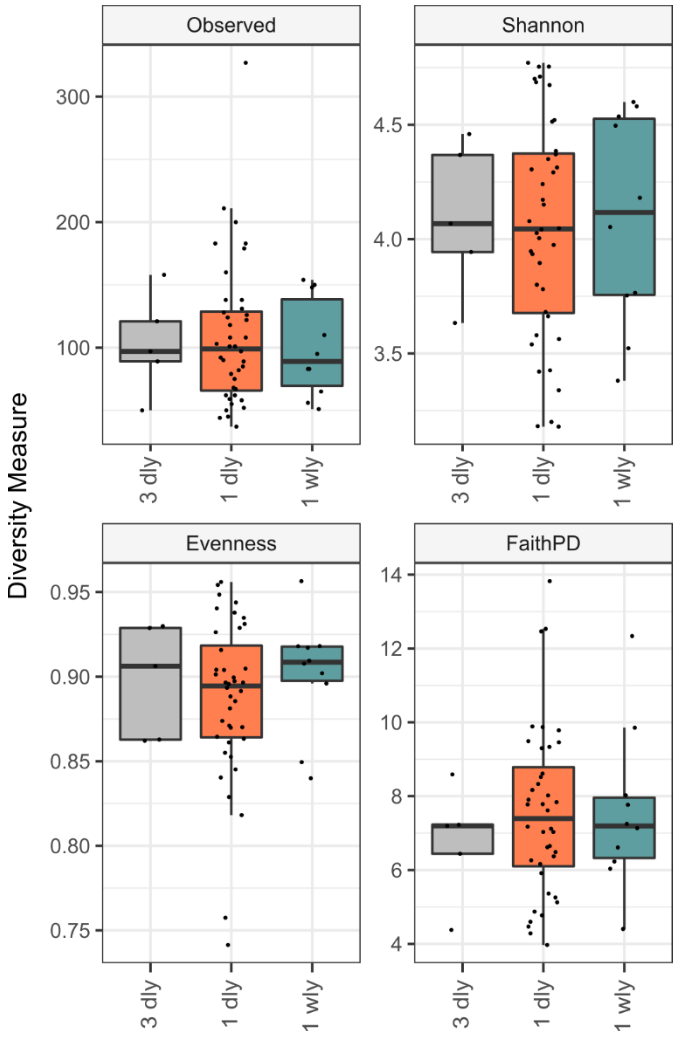

Supplementary Figure 2: Comparison of alpha diversity measures between different cleaning intervals within TC1 (3dly = cleaning three times a day: n = 5, 1dly = daily cleaning: n = 41, 1wly = weekly cleaning: n = 10). Differences are shown by four indices (observed taxonomic units, Shannon, Pielou's Evenness, Faith PD diversity index). Points represent individual samples. Displayed are the median, the 25% and 75% quartiles and outliers. Whiskers represent the lowest and highest microbial counts within the 1.5-fold of the interquartile range (IQR) (the 25% and 75% quartile). No differences were found statistically significant ($p > 0.05$). TC = Tertiary Center.

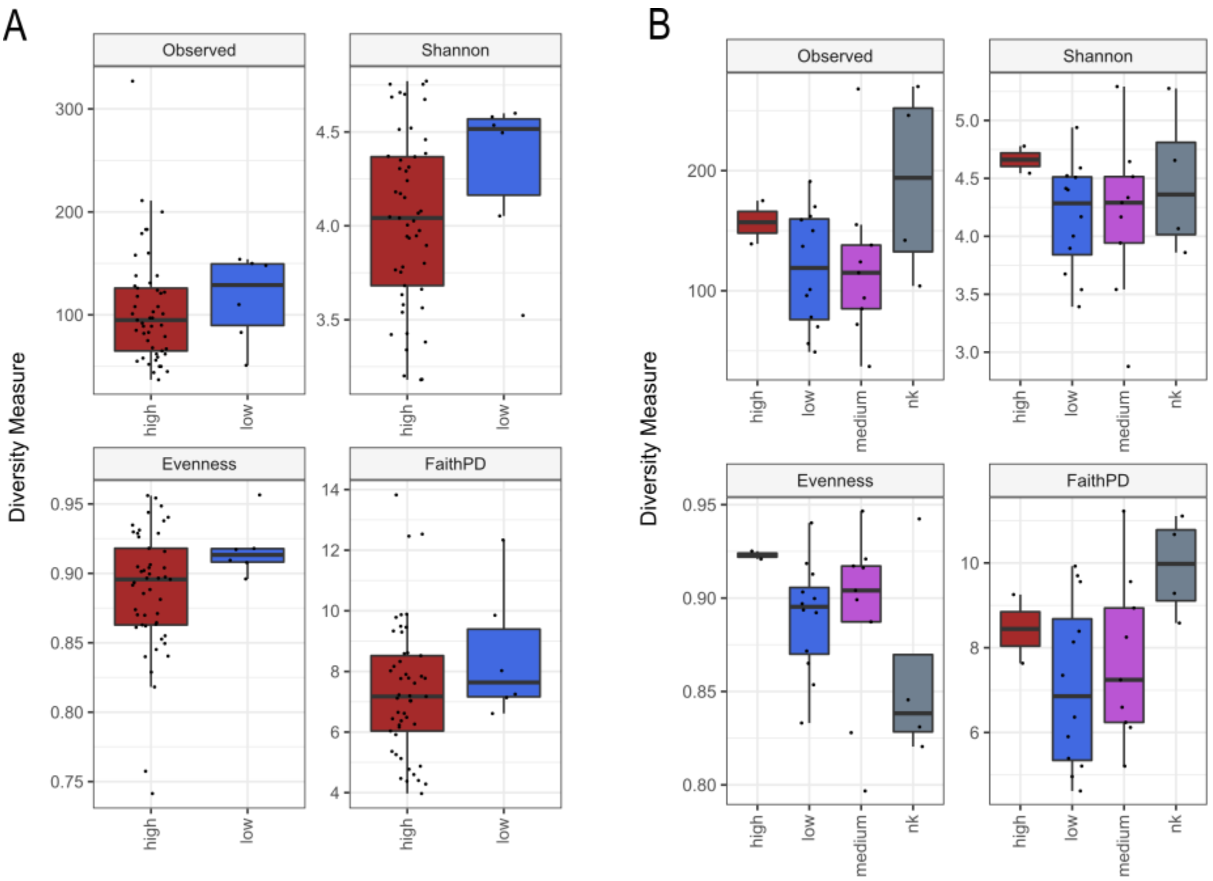

Supplementary Figure 3: Comparison of alpha diversity measures between samples with different patient throughput in TC1 (A) and TC2 (B) (high = 70 – 200, TC 1: n = 50, TC 2: n = 2; medium = 10-70, TC 2: n = 9; low = 1-10, TC 1: n = 8, TC 2: n = 12; nk = not known, no occupancy data were provided, TC 2: n = 4). Differences are shown by four indices (observed taxonomic units, Shannon, Pielou's Evenness, Faith PD diversity index). Points represent individual samples. Displayed are the median, the 25% and 75% quartiles and outliers. Whiskers represent the lowest and highest microbial counts within the 1.5-fold of the interquartile range (IQR) (the 25% and 75% quartile). No differences were found statistically significant ($p > 0.05$). TC = Tertiary Center.

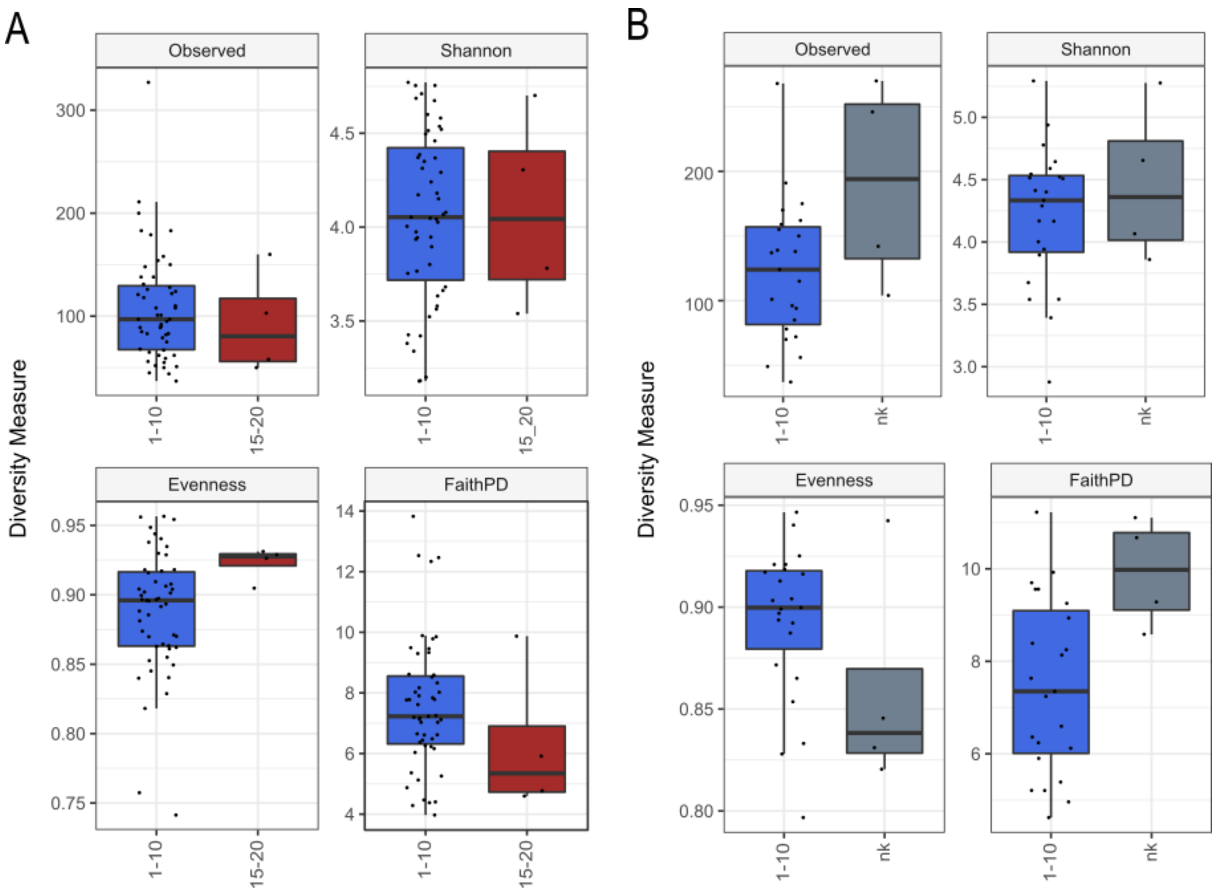

Supplementary Figure 4: Comparison of alpha diversity measures between samples with different occupations of physicians in TC1 (A) and TC2 (B) (1-10, TC1: n = 52, TC2: n = 9; 15-20, TC1: n = 4; nk = no occupancy data were provided, TC2: n = 4). Differences are shown by four indices (observed taxonomic units, Shannon, Pielou's Evenness, Faith PD diversity index). Points represent individual samples. Displayed are the median, the 25% and 75% quartiles and outliers. Whiskers represent the lowest and highest microbial counts within the 1.5-fold of the interquartile range (IQR) (the 25% and 75% quartile). No differences were found statistically significant ($p > 0.05$). TC = Tertiary Center.

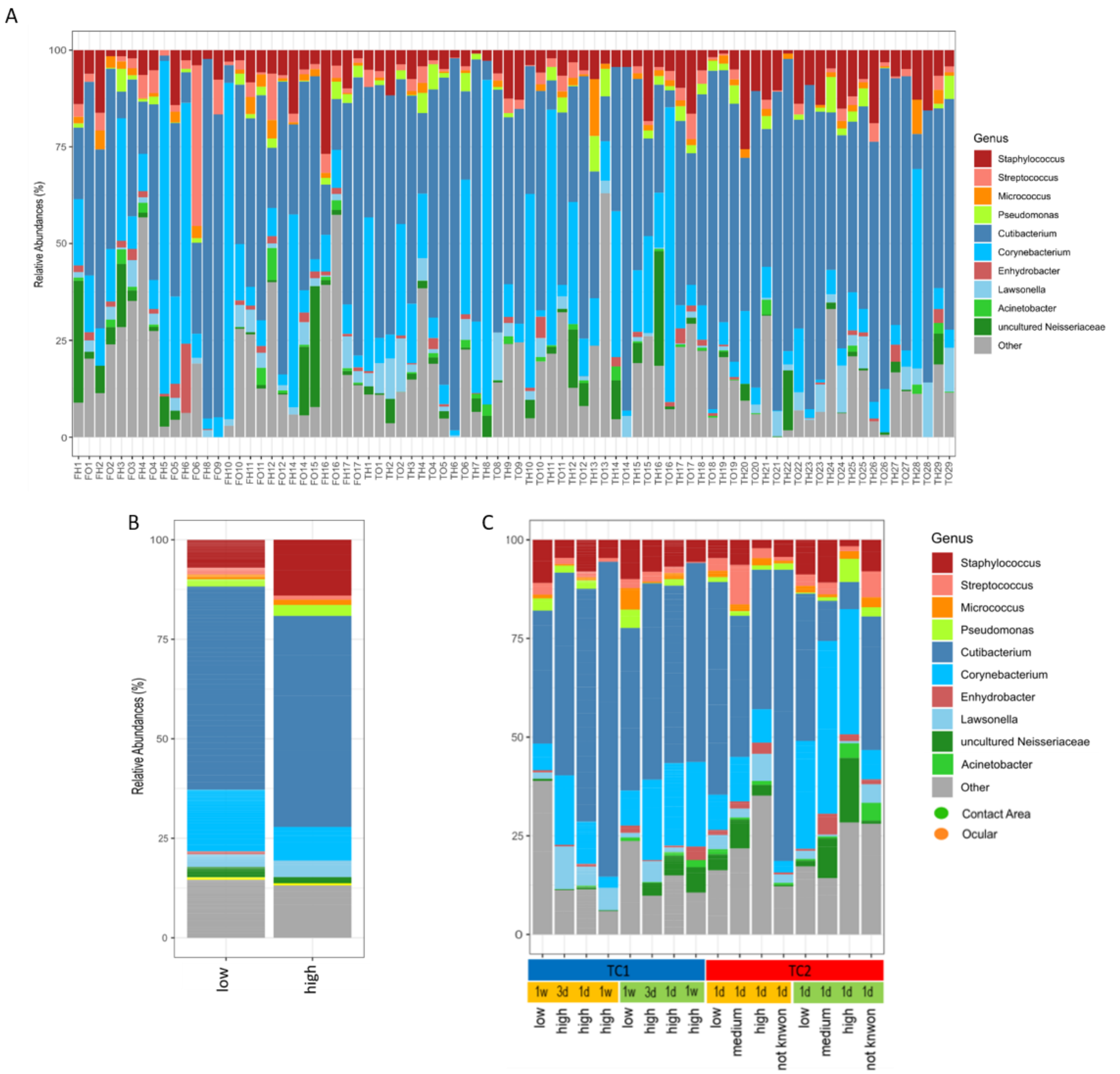

Supplementary Figure 5: Bar chart of relative abundances of ASVs (amplicon sequence variants) classified on genus level. A) Bars represent single slit lamp samples. B) Taxonomic composition of the slit lamp bacteriota from TC1 aggregated to the physicians occupancy (low = 1-10 physicians / day; high = 10 - 20 physicians / day). No data could be obtained from TC 2. C) Taxonomic composition of the slit lamp bacteriota from TC1 and TC2 respectively, and the respective sample sites (orange = ocular samples; green = contact area samples). Displayed are also patient occupancy (low = 1-10 patients / day; medium = 10 – 70 patients / day; high = 70 – 200 patients / day) and cleaning intervals (1w = weekly, 1d = daily; 3d = three times a day). In both clinics, all slit lamp contact areas were claimed to be wipe disinfected between different patients. More comprehensive cleaning data were obtained from TC1. Here, slit lamps were in addition cleaned carefully either three times a day, once a day or weekly. TC = Tertiary Center. Taxa with relative abundance of less than 1% were collectively summarized as ‘Other’. T = TC1, F = TC2; H = Contact Area, O =Ocular. TC = Tertiary Center.

Supplementary Table 1: Metadata for all samples; Occ_AggrPat = Patiens Occupancy, OcupancyMed = Physicians Occupancy; 3_dly = three time a day; 1_dly = daily, 1_wly = weekly, nk = not known

SampleID	Location	Type	RoomNr.	OcupancyPatients	Occ_AggrPat	OcupancyMed	Cleaning
TO1	Tuebingen	Ocular	5372	70	high	6	3_dly
TH1	Tuebingen	ContactArea	5372	70	high	6	3_dly
TO2	Tuebingen	OcularWindow	5372	70	high	6	3_dly
TH2	Tuebingen	ContactAreaWindow	5372	70	high	6	3_dly
TO3	Tuebingen	Ocular	5373	70	high	6	3_dly
TH3	Tuebingen	ContactArea	5373	70	high	6	3_dly
TO4	Tuebingen	Ocular	5141	140	high	2	1_dly
TH4	Tuebingen	ContactArea	5141	140	high	2	1_dly
TO5	Tuebingen	Ocular	5140	200	high	3	1_dly
TH5	Tuebingen	ContactArea	5140	200	high	3	1_dly
TO6	Tuebingen	Ocular	5144	70	high	2	1_dly
TH6	Tuebingen	ContactArea	5144	70	high	2	1_dly
TO7	Tuebingen	Ocular	5145	70	high	2	1_dly
TH7	Tuebingen	ContactArea	5145	70	high	2	1_dly
TO8	Tuebingen	Ocular	5146	120	high	2	1_dly
TH8	Tuebingen	ContactArea	5146	120	high	2	1_dly
TO9	Tuebingen	Ocular	5120	10	low	2	1_wly
TH9	Tuebingen	ContactArea	5120	10	low	2	1_wly
TO10	Tuebingen	Ocular	4372	70	high	6	1_dly
TH10	Tuebingen	ContactArea	4372	70	high	6	1_dly
TO11	Tuebingen	OcularWindow	4372	70	high	6	1_dly
TH11	Tuebingen	ContactAreaWindow	4372	70	high	6	1_dly
TO12	Tuebingen	Ocular	4373	70	high	6	1_dly
TH12	Tuebingen	ContactArea	4373	70	high	6	1_dly
TO13	Tuebingen	Ocular	4119	4	low	1	1_wly
TH13	Tuebingen	ContactArea	4119	4	low	1	1_wly
TO14	Tuebingen	Ocular	4131	150	high	10	1_wly
TH14	Tuebingen	ContactArea	4131	150	high	10	1_wly
TO15	Tuebingen	Ocular	4113	100	high	1	1_dly
TH15	Tuebingen	ContactArea	4113	100	high	1	1_dly
TO16	Tuebingen	Ocular	4112	100	high	2	1_dly
TH16	Tuebingen	ContactArea	4112	100	high	2	1_dly
TO17	Tuebingen	Ocular	3102	10	low	2	1_wly
TH17	Tuebingen	ContactArea	3102	10	low	2	1_wly
TO18	Tuebingen	Ocular	3154	70	high	2	1_dly
TH18	Tuebingen	ContactArea	3154	70	high	2	1_dly
TO19	Tuebingen	Ocular	3156	70	high	2	1_dly
TH19	Tuebingen	ContactArea	3156	70	high	2	1_dly
TO20	Tuebingen	Ocular	3207	120	high	15	1_dly
TH20	Tuebingen	ContactArea	3207	120	high	15	1_dly
TO21	Tuebingen	Ocular	3209	110	high	10	1_dly
TH21	Tuebingen	ContactArea	3209	110	high	10	1_dly
TO22	Tuebingen	Ocular	3210	110	high	10	1_dly
TH22	Tuebingen	ContactArea	3210	110	high	10	1_dly
TO23	Tuebingen	Ocular	3212	110	high	10	1_dly
TH23	Tuebingen	ContactArea	3212	110	high	10	1_dly
TO24	Tuebingen	Ocular	3206	150	high	20	1_dly
TH24	Tuebingen	ContactArea	3206	150	high	20	1_dly
TO25	Tuebingen	Ocular	3214	110	high	10	1_dly
TH25	Tuebingen	ContactArea	3214	110	high	10	1_dly
TO26	Tuebingen	Ocular	3155	70	high	2	1_dly
TH26	Tuebingen	ContactArea	3155	70	high	2	1_dly
TO27	Tuebingen	Ocular	3241	110	high	3	1_wly
TH27	Tuebingen	ContactArea	3241	110	high	3	1_wly
TO28	Tuebingen	Ocular	3211	110	high	10	1_dly
TH28	Tuebingen	ContactArea	3211	110	high	10	1_dly
TO29	Tuebingen	Ocular	3213	110	high	10	1_dly
TH29	Tuebingen	ContactArea	3213	110	high	10	1_dly
FO1	Freiburg	Ocular	U01	184	high	20	nk
FH1	Freiburg	ContactArea	U01	184	high	20	nk
FO2	Freiburg	Ocular	U02	128	high	10	nk
FH2	Freiburg	ContactArea	U02	128	high	10	nk
FO3	Freiburg	Ocular	U03	85	high	9	nk
FH3	Freiburg	ContactArea	U03	85	high	9	nk
FO4	Freiburg	Ocular	U04	96	high	9	nk

SampleID	Location	Type	RoomNr.	OcupancyPatients	Occ_AggrPat	OcupancyMed	Cleaning
FH4	Freiburg	ContactArea	U04	96	high	9	nk
FO5	Freiburg	Ocular	U05	90	high	8	nk
FH5	Freiburg	ContactArea	U05	90	high	8	nk
FO6	Freiburg	Ocular	U06	64	medium	8	nk
FH6	Freiburg	ContactArea	U06	64	medium	8	nk
FO7	Freiburg	Ocular	U07	85	high	10	nk
FH7	Freiburg	ContactArea	U07	85	high	10	nk
FO8	Freiburg	Ocular	U08	81	high	10	nk
FH8	Freiburg	ContactArea	U08	81	high	10	nk
FO9	Freiburg	Ocular	U09	47	medium	8	nk
FH9	Freiburg	ContactArea	U09	47	medium	8	nk
FO10	Freiburg	Ocular	U010	10	low	5	nk
FH10	Freiburg	ContactArea	U010	10	low	5	nk
FO11	Freiburg	Ocular	U011	2	low	1	nk
FH11	Freiburg	ContactArea	U011	2	low	1	nk
FO12	Freiburg	Ocular	Fotografie	nk	nk	nk	nk
FH12	Freiburg	ContactArea	Fotografie	nk	nk	nk	nk
FO13	Freiburg	Ocular	L1	10	low	6	nk
FH13	Freiburg	ContactArea	L1	10	low	6	nk
FO14	Freiburg	Ocular	L2	6	low	4	nk
FH14	Freiburg	ContactArea	L2	6	low	4	nk
FO15	Freiburg	Ocular	OP	21	medium	4	nk
FO16	Freiburg	Ocular	SeH	18	medium	4	nk
FH16	Freiburg	ContactArea	SeH	18	medium	4	nr
FO17	Freiburg	Ocular	U12	nk	nk	nk	nr
FH17	Freiburg	ContactArea	U12	nk	nk	nk	nr
L1	Control	EmptySwab	ControlNeg	nr	nr	nr	nr
L2	Control	EmptySwab	ControlNeg	nr	nr	nr	nr
MO1	Control	MockV3V4	ControlPos	nr	nr	nr	nr
MO2	Control	MockV3V4	ControlPos	nr	nr	nr	nr
MOR	Control	MockV3V4	ControlPos	nr	nr	nr	nr

Supplementary Table 2: Relatively most abundant genera, identified in a combined dataset of TC1 and TC2 and selected representatives at the species level, known to be affiliated with eye diseases.

**SILVA genus**	**Potentially pathogenic species**	**RG***	**Origin**	**Eye disease**
Cutibacterium	*Cutibacterium acnes*	2	skin	endophtalmitis[1,2]
Corynebacterium	*Corynebacterium sp*	n.a.	environment / skin / mucosa	different species known to cause endophthalmitis, conjunctivitis[3,4]
Staphylococcus	*Staphylococcus epidermidis*	2	skin	conjunctivitis, endophthalmitis[5]
Staphylococcus	*Staphylococcus hominis*	2	skin / axillae / pubic	endophtalmitis[6]
Staphylococcus	*Staphylococcus aureus*	2	environment / skin	blepharitis, endophthalmitis, bacterial conjunctivitis[7,8]
uncultured *Neisseriaceae*	*Neisseria gonorrhoeae*	2	urogenital tract	gonococcal conjunctivitis[8]
Streptococcus	*Streptococcus pneumoniae*	2	respiratory tract	bacterial keratitis[8]
Pseudomonas	*Pseudomonas aeruginosa*	2	ubiquitous	bacterial keratitis[9]
Micrococcus	*Micrococcus luteus / sp.*	1	environment, skin	endophtalmitis[10], keratitis[11]
Enhydrobacter	*Enhydrobacter sp.*	n.a.	skin / mucosa / respiratory tract	higher shares at blepharitis patients[12]

Only the genera with an overall relative abundance of > 1% and known to be affiliated with eye diseases are displayed. *RG = risk group classification according to German TRBA 466 [13]; n.a. = not applicable, as risk group classification is species-specific. TC = Tertiary Center.

References

1. Cogen, A. L., Nizet, V. & Gallo, R. L. (2008). Skin microbiota: a source of disease or defence? *Br J Dermatol* 158, 442–455; doi.10.1111/j.1365-2133.2008.08437.x

2. Ovodenko, B., Seedor, J., Ritterband, D., Sha, M., Yang, R., Koplin, R. (2009). The prevalence and pathogenicity of Propionibacterium acnes keratitis. *Cornea* 28, 36–39; doi.10.1097/ICO.0b013e3181839b1a.

3. Kuriyan, A. E., Sridhar, J., Flynn, H.W., Huang, L.C., Yannuzzi, N.A., Smiddy, W.E. *et al.*(2017). Endophthalmitis Caused by Corynebacterium Species: Clinical Features, Antibiotic Susceptibility, and Treatment Outcomes. *Ophthal Retina* 1, 200–205; doi.10.1016/j.oret.2016.11.007.

4. Hinić, V., Lang, C., Weisser, M., Straub, C., Frei, R., Goldenberger, D. (2012). Corynebacterium tuberculostearicum: a Potentially Misidentified and Multiresistant Corynebacterium Species Isolated from Clinical Specimens. *J Clin Microbiol* 50, 2561–2567; doi.10.1128/JCM.00386-12.

5. Flores-Páez, L. A., Zenteno, J.C., Alcántar-Curiel, M.D., Vargas-Medoza, C.F., Rodríguez-Martínez, S., Cancino-Diaz, M.E. *et al.* (2015). Molecular and Phenotypic Characterization of Staphylococcus epidermidis Isolates from Healthy Conjunctiva and a Comparative Analysis with Isolates from Ocular Infection. *PLoS ONE* 10, e0135964; doi.10.1371/journal.pone.0135964.

6. Wong, R. W. & Rhodes, K. M. (2015). Endophthalmitis caused by Staphylococcus hominis and two different colonies of Staphylococcus haemolyticus after cataract surgery. *Retin Cases Br Rep* 9, 181–184; doi.10.1097/ICB.0000000000000133 .

7. O'Callaghan, R. J. (2018). The Pathogenesis of Staphylococcus aureus Eye Infections. *Pathogens* 7; doi.10.3390/pathogens7010009.

8. Watson, S., Cabrera-Aguas, M. & Khoo, P. (2018). Common eye infections. *Austr Prescr* 41, 67–72; doi.10.18773/austprescr.2018.016.

9. Stern, G. A. (1990). Pseudomonas keratitis and contact lens wear: the lens/eye is at fault. *Cornea* 9 Suppl 1, S36-8; discussion S39-40; doi.0.1097/00003226-199010001-00015.

10. Cartwright, M. J., King, M. H., Weinberg, R. S. & Guerry, R. K. (1990). Micrococcus endophthalmitis. *Arch Ophthalmol* 108, 1523–1524; doi.10.1001/archopht.1990.01070130025012.

11. Taneja, M., Rathi, V.M., Bagga, B., Murthy, S.I., Shar, J., Reddy, A.K. *et al.* (2019). Micrococcus keratitis following microkeratome-assisted laser in situ keratomileusis. *Oman J Ophthalmol* 12, 203–205; doi.10.4103/ojo.OJO_54_2017.

12. Lee, S. H., Oh, D. H., Jung, J. Y., Kim, J. C. & Jeon, C. O. (2012). Comparative ocular microbial communities in humans with and without blepharitis. *Invest Ophthalmol Vis Sci* 53, 5585–5593; doi.10.1167/iovs.12-9922.

13. Technical Rules for Biological Agents: Classification of prokaryotes (bacteria and archaea) into risk groups. TRBA 466. Available at, https://www.baua.de/EN/Service/Legislative-texts-and-technical-rules/Rules/TRBA/TRBA-466.html
